# Echocardiographic Assessment of Ebstein's Anomaly in a 60-Year-Old Man

**DOI:** 10.1155/2009/653741

**Published:** 2009-06-07

**Authors:** Elisabetta Palmerini, Duccio Federici, Alessia Del Pasqua, Sonia Bernazzali, Matteo Lisi, Mario Chiavarelli, Sergio Mondillo

**Affiliations:** Department of Cardiovascular Diseases, University Hospital “S. Maria le Scotte”, 53100 Siena, Italy

## Abstract

We present an echocardiographic evaluation of an elderly man affected with Ebstein's anomaly. In the natural history of this congenital disease only 5% of patients survive beyond the fifth decade. The patient presented severe right heart failure and he was refered to our institution for heart transplantation.

## 1. Introduction

Ebstein's anomaly is a rare congenital heart disorder occurring in ≈ 1 per 200.000 live births and accounting for <1% of all congenital heart diseases [1]; only 5% of patients survive beyond the fifth decade without surgical correction [2]. The disorder consists of a tricuspid valve and right ventricle malformation characterized by a downward apical displacement of the septal and posterior tricuspid leaflets, adherence of the septal and posterior leaflets to the underlying myocardium due to a failure of the embryologic delamination process (or splitting) of the issue by detachment of the inner layer, dilatation of the “atrialized” and “true” right ventricle with development of hypertrophy and thinning of the wall. In normal human heart the downward displacement of the septal and posterior tricuspid valve leaflets is <8 mm/m^2^ body surface area from the insertion of the anterior mitral valve leaflet [3]. 

The tricuspid anterior leaflet may be severely deformed: frequently the only mobile leaflet tissue is displaced into the right ventricular outflow tract, where it may cause obstruction or form a large sail-like intracavitary curtain. It appears redundant sometimes with fenestrations; the right atrioventricular junction (or true tricuspid annulus) appears enlarged. Its chordae tendineae are generally short and poorly formed [4].

## 2. Case Description

We present an echocardiographic assessment of a Caucasian 60-years old man suffering from Ebstein's anomaly referred to our institution for heart transplantation evaluation. 

The diagnosis of Ebstein's anomaly was done when he was 40 years old and he was not initially referred for surgery. Actually he presents with signs and symptoms of right heart failure (pleural effusion, hepatomegaly, low extremities edemas), reduced exercise tolerance and effort dyspnea (NYHA class III-IV). He recently has shown worsening of his clinical status with episodes of substained ventricular tachycardia requiring ICD/DDD implantation. Because of advanced cardiac dysfunction, corrective or palliative surgical procedures were excluded and the patient underwent heart transplantation evaluation. 

**Figure 1:**
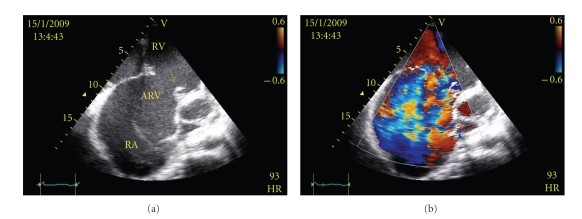
Transthoracic apical four-chamber view showing: (a) downward displacement of the septal leaflet of the tricuspid valve (arrow) in relation to the anterior mitral valve leaflet (MV), several enlargement of right atrium (RA), atrialized portion of right ventricle (ARV), and true right ventricle (RV); (b) severe tricuspid valve regurgitation due to the anomalous valvular arrangement.

**Figure 2:**
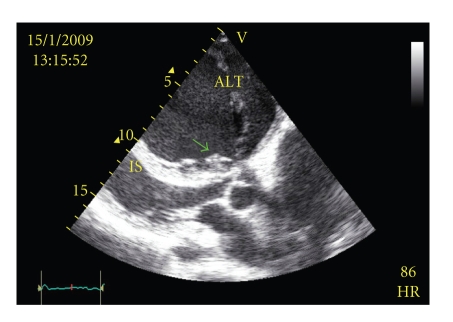
Transthoracic parasternal long-axis view demonstrating the septal leaflet apical displacement of the tricuspid valve (arrow), the “sail-like configuration” of the enlarged anterior tricuspid leaflet (ATL), and the leftward shift of the interventricular septum reducing left ventricle compliance (IS).

The images in (Figures 1, 2) show that the characteristic apical displacement of septal tricuspid valve leaflet is 30 mm from the insertion of the anterior mitral valve leaflet. Marked enlargement of the right chambers and severe tricuspid regurgitation are also present and the left ventricle too appears dysfunctional.

Considering the rarity of Ebstein's anomaly in elderly it is important to point out the central role of echocardiography in the assessment of the morphological anatomy, the right ventricle dysfunction followup and the planning of the best surgical procedure.
